# Carbon Monitor Europe near-real-time daily CO_2_ emissions for 27 EU countries and the United Kingdom

**DOI:** 10.1038/s41597-023-02284-y

**Published:** 2023-06-08

**Authors:** Piyu Ke, Zhu Deng, Biqing Zhu, Bo Zheng, Yilong Wang, Olivier Boucher, Simon Ben Arous, Chuanlong Zhou, Robbie M. Andrew, Xinyu Dou, Taochun Sun, Xuanren Song, Zhao Li, Feifan Yan, Duo Cui, Yifan Hu, Da Huo, Jean-Pierre Chang, Richard Engelen, Steven J. Davis, Philippe Ciais, Zhu Liu

**Affiliations:** 1 Department of Earth System Science, Tsinghua University, Beijing, China; 2 Department of Mathematics and Statistics, Faculty of Environment, Science and Economy, University of Exeter, Exeter, UK; 3 Alibaba Cloud, Hangzhou, China; 4 Laboratoire des Sciences du Climate et de l’Environnement LSCE, Orme de Merisiers, 91191 Gif-sur-Yvette, France; 5 Institute of Environment and Ecology, Tsinghua Shenzhen International Graduate School, Tsinghua University, Shenzhen, 518055 China; 6 Key Laboratory of Land Surface Pattern and Simulation, Institute of Geographical Sciences and Natural Resources Research, Chinese Academy of Sciences, Beijing, 100101 China; 7 Institute Pierre-Simon Laplace, Sorbonne Université/CNRS, Paris, France; 8 Kayrros, 33 Rue La Fayette, 75009 Paris, France; 9 CICERO Center for International Climate Research, Oslo, 0349 Norway; 10 Key Laboratory of Marine Environment and Ecology, and Frontiers Science Center for Deep Ocean Multispheres and Earth System, Ministry of Education, Ocean University of China, Qingdao, 266100 China; 11 Key Laboratory of Sustainable Forest Ecosystem Management, Northeast Forestry University, Harbin, 150040 China; 12 Department of Civil & Mineral Engineering, University of Toronto, Toronto, ON M5S 1A4 Canada; 13 CITEPA, 42 Rue de Paradis, 75010 Paris, France; 14 European Centre for Medium-Range Weather Forecasts, Reading, RG2 9AX UK; 15 Department of Earth System Science, University of California, Irvine, 3232 Croul Hall, Irvine, CA 92697-3100 USA; 16 Climate and Atmosphere Research Center (CARE-C) The Cyprus Institute 20 Konstantinou Kavafi Street, 2121 Nicosia, Cyprus

**Keywords:** Climate change, Environmental sciences

## Abstract

With the urgent need to implement the EU countries pledges and to monitor the effectiveness of Green Deal plan, Monitoring Reporting and Verification tools are needed to track how emissions are changing for all the sectors. Current official inventories only provide annual estimates of national CO_2_ emissions with a lag of 1+ year which do not capture the variations of emissions due to recent shocks including COVID lockdowns and economic rebounds, war in Ukraine. Here we present a near-real-time country-level dataset of daily fossil fuel and cement emissions from January 2019 through December 2021 for 27 EU countries and UK, which called Carbon Monitor Europe. The data are calculated separately for six sectors: power, industry, ground transportation, domestic aviation, international aviation and residential. Daily CO_2_ emissions are estimated from a large set of activity data compiled from different sources. The goal of this dataset is to improve the timeliness and temporal resolution of emissions for European countries, to inform the public and decision makers about current emissions changes in Europe.

## Background & Summary

The European Union and the United Kingdom is the world’s third energy consumer and CO_2_ emitter, accounting for 12% of global emissions^1–3^. The EU27 announced the Green Deal in December 2019, and set up a road map for cutting greenhouse gas emissions by at least 55% by 2030 and reaching carbon neutrality by 2050^4^. With increasing focus and effort on reducing CO_2_ emissions, there is a growing need for more reliable data as well as for data with a lower latency. Most CO_2_ emission inventories including national inventories reported to the United Nations Framework Convention on Climate Change (UNFCCC) lag reality by one years or more^2,5–10^.

Lower latency estimates of CO_2_ emissions are generally delayed by several months as data must be gathered from numerous sources and then verified. For example, Eurostat has been producing early estimates of annual and country-level CO_2_ emissions in the EU since at least 2012, with a delay for about 5 months^11^. The Global Carbon Project publishes projections of the current year’s global fossil CO_2_ emissions since 2012 by the end of the current year^12^. Few EU countries and the UK publish quarterly or monthly estimates of emissions from preliminary energy data^13–17^. As the COVID-19 pandemic profoundly disrupted human activities in the year 2020, existing inventories were not able to monitor changes in activity and assess the COVID-19 impacts on CO_2_ emissions during that period and the following recovery. Le Quéré *et al*.^18^ and Forster *et al*.^19^ developed methods for estimating daily, global CO_2_ emissions, based on confinement index and Google mobility indexes respectively^18,19^. These two approaches captured first order pandemic related reductions but were not tested for subsequent variations when lockdowns were finished, and did not continue to provide near-real-time, daily and country-level estimates emissions. Further, the private company Kayrros released a daily estimate of emissions from regulated sectors (European Trading Scheme) in November 2021 using site-level satellite monitoring of industrial activity, called Carbon Watch^20^. The Carbon Monitor international research initiative developed a new near-real-time daily dataset of CO_2_ emissions with global coverage and country-level estimates for 12 countries which lags reality by only one month^1,3,21–26^. Building on this foundation, they have also developed the Carbon Monitor China^27^, which provides provincial-level near-real-time daily emissions data for China, and the Carbon Monitor United States^28^, which provides state-level daily emissions data for the US. Additionally, they have launched the Carbon Monitor Cities^29,30^, covering near-real-time daily emissions data for over 1500 cities worldwide, the Carbon Monitor Power^31^, which provides near-real-time hourly-to-daily data on power generation, and the Carbon Monitor GRACED^32,33^, a dataset of global near-real-time grid-level CO_2_ emissions.

Here, we present a near-real-time, sector-specific, country-level estimates of daily fossil fuel and cement CO_2_ emissions for 27 European Union countries and the United Kingdom based on an extension of Carbon Monitor called Carbon Monitor Europe (CM-EU). We present the methodology and the results for changes in emissions between January 1, 2019 and December 31, 2021. Details of our data sources and approach are in the Methods section. An evaluation of CM-EU against national quarterly emissions estimates and Carbon Watch is provided in the technical validation section. The data Carbon Monitor Europe are publicly available at Figshare^34^ and our website https://eu.carbonmonitor.org/.

## Methods

Carbon Monitor Europe (CM-EU) is a new regional improved version of the global Carbon Monitor system, providing near-real-time daily estimates of CO_2_ emissions for six sectors over the globe with separate estimates for 12 countries or groups of countries^1,21^. CM-EU presents country-level estimates of daily CO_2_ emissions from January 2019 through December 2021 for 27 countries of European Union (EU27) and the United Kingdom (UK). Following the method presented in the global Carbon Monitor publications^1,21^, we used annual CO_2_ emissions of EU27 & UK from the Emissions Database for Global Atmospheric Research (EDGAR)^8^ as the baseline data for emissions in the year 2019, then disaggregated and extrapolated in time this estimate into daily scale for 2020 and 2021 based on time variations of activity data. Below we describe the calculation process in detail.

### Annual country-level and sectoral CO_2_ emissions in the baseline year 2019

Annual fossil fuel combustion and cement production CO_2_ emissions by sector in 2019 for all European Union countries and United Kingdom are directly obtained from the *Fossil CO*_*2*_ *emissions of all world countries - 2020 Report*^8^ released by the Emissions Database for Global Atmospheric Research (EDGAR). Emissions of EDGAR are derived using the IPCC Tier 1 approach according to the 2006 IPCC Guidelines^35^, with 35 sectors based on IPCC categories. We aggregated 11 energy-related and cement production sectors of EDGAR into six main sectors, including power, industry, ground transportation, residential (public, commercial buildings and households), domestic and international aviation. In this dataset, emissions from international flights are assigned to the country of departure, which is different from the accounting used by UNFCCC from IPCC guidelines where international flights are assigned to a specific bunker fuel category. Table 1 shows the correspondence table between aggregated sectors of CM-EU and EDGAR sectors based on IPCC (first column).

**Table 1:** The mapping relationships between sectors in this study and EDGAR sectors based on IPCC categories.

IPCC code	Sectors of EDGAR	Sectors of CM-EU
1A1a	Public electricity and heat production	Power
1A1bc	Other energy industries	Industry (incl. cement production)
1A2	Manufacturing industries and construction	
2A1	Cement production	
1A3a	Domestic aviation	Domestic aviation
1A3b	Road transportation no resuspension	Ground transportation
1A3c	Rail transportation	
1A3d	Inland navigation	
1A3e	Other transportation	
1A4	Residential and other sectors	Residential
1C1	Memo: International aviation (bunker fuels)	International aviation

### Data acquisition and processing of daily CO_2_ emissions in 2019, 2020 and 2021

Carbon Monitor follows the IPCC guidelines for emissions reporting^35^ in computing CO_2_ emissions from a country/region by multiplying activity data (AD) by corresponding emissions factors (EF):

$$Emis=\sum \sum \sum A{D}_{i,j,k}\times E{F}_{i,j,k}$$

where i, j, k denote regions, sectors, and fuel types respectively. We assume that emission factors and structure of each sector remain unchanged for each country in 2020 and 2021 compared with 2019. Thus, the rate of change of the emission is calculated based solely on the change of the activity data in 2020 and 2021 compared to the same period of 2019. The emissions were calculated following this equation separately for the power sector, the industrial sector, the ground transportation sector, the aviation sector (including the domestic and international aviation sector) and the residential sector.

#### Power sector

For the power sector, daily emissions were calculated from hourly electricity generation data by production types at resolution of 1 h to 15 min. Data of 22 EU countries (Austria, Belgium, Bulgaria, Croatia, Cyprus, Czech Republic, Denmark, Estonia, Finland, France, Germany, Greece, Hungary, Ireland, Italy, Latvia, Netherlands, Poland, Portugal, Slovakia, Slovenia and Spain) and United Kingdom are available from the ENTSO-E Transparency platform (https://transparency.entsoe.eu/dashboard/show) and Balancing Mechanism Reporting Service (BMRS) (https://www.bmreports.com/) respectively (Table 2). We removed outliers and filled the N/A values by using the linear interpolation function in Python Pandas packages. The electricity generation data used in this study from ENTSO-E and BMRS are include several fuel types with coal, gas, oil and peat. The average yearly emission factor of the whole power sector is assumed to remain constant at its 2019 value, so that daily emissions are estimated by:

$$Emi{s}_{power,daily,2019}=Emi{s}_{power,yearly,2019}\times \frac{A{D}_{power,daily,2019}}{A{D}_{power,yearly,2019}}$$

$$Emi{s}_{power,daily,2020or2021}=Emi{s}_{power,daily,2019}\times \frac{A{D}_{power,daily,2020or2021}}{A{D}_{power,daily,2019}}$$

**Table 2:** Sources of activity data for different sectors.

Sector	Country	Data type	Data source
Power	EU27	Hourly thermal production	ENTSO-E Transparency platform (https://transparency.entsoe.eu/dashboard/show)
	UK	Hourly power generation	Balancing Mechanism Reporting Service (BMRS) (https://www.bmreports.com/)
Industry	EU27 (exc. Ireland)	Industrial Production Index (IPI)	Eurostat (https://ec.europa.eu/eurostat)
	Ireland	Industrial Production Index (IPI)	Central Statistics Office (https://data.cso.ie)
	UK	Industrial Production Index (IPI)	Office for National Statistics (https://www.ons.gov.uk)
Ground Transportation	EU27 + UK	TomTom Congestion Level	TomTom Traffic Index (https://www.tomtom.com/en_gb/traffic- index/)
Residential	EU27 + UK	Population-weighted heating degree days	ERA5 reanalysis of 2-meters air temperature (Copernicus Climate Change Service (C3S), 2019)
Aviation	EU27 + UK	Flight distance	FlightRadar24 (https://www.flightradar24.com/)

For Lithuania, Luxembourg, Malta, Romania and Sweden (other EU) not covered by ENSTO-E, we assume a linear relationship between their daily emissions and the total daily emissions of the 22 EU countries and United Kingdom (EU22 + UK) and their daily emissions are estimated as:

$$Emi{s}_{power,daily,2019,EU5}=Emi{s}_{power,yearly,2019,otherEU}\times \frac{Emi{s}_{power,daily,2019,EU22+UK}}{Emi{s}_{power,yearly,2019,EU22+UK}}$$

$$Emi{s}_{power,daily,2020or2021,otherEU}=Emi{s}_{power,daily,2019,EU5}\times \frac{Emi{s}_{power,daily,2020or2021,EU22+UK}}{Emi{s}_{power,daily,2019,EU22+UK}}$$

#### Industry sector

Daily emissions from industry are estimated using the monthly industrial production index (IPI) from several datasets and daily power generation data (Table 2). As daily production data are not available for industrial and cement production, the monthly CO_2_ emissions are estimated by using monthly statistics of industrial production and daily data of electricity generation to disaggregate the monthly CO_2_ emissions into a daily scale. This approach is based on two assumptions: 1. A linear relationship is assumed between industrial production index and emissions from industrial and cement production. 2. A linear relationship is assumed between daily industry activity and daily electricity production, from ENSTO-E and our approach for the five Other-EU countries. Therefore, the monthly and daily industry emissions are estimated following:

$$Emi{s}_{industry,monthly,2019}=Emi{s}_{industry,yearly,2019}\times \frac{A{D}_{industry,monthly,2019}}{A{D}_{industry,yearly,2019}}$$

$$Emi{s}_{industry,monthly,2020or2021}=Emi{s}_{industry,monthly,2019}\times \frac{A{D}_{industry,monthly,2020or2021}}{A{D}_{industry,monthly,2019}}$$

$$Emi{s}_{industry,daily,2019,2020or2021}=Emi{s}_{industry,monthly,2019,2020or2021}\times \frac{A{D}_{power,daily,2019,2020or2021}}{A{D}_{power,monthly,2019,2020or2021}}$$

#### Ground transportation sector

Carbon Monitor uses TomTom congestion global level data (Table 2) from the TomTom website (https://www.tomtom.com/en_gb/traffic-index/) to capture the daily variations in the ground transportation activity. The TomTom traffic congestion level (called G hereafter) represents the extra time spent on a trip in congested conditions, as a percentage, compared to uncongested conditions. TomTom congestion level data cover 203 cities across 24 EU countries (Austria, Belgium, Bulgaria, Czech Republic, Denmark, Estonia, Finland, France, Germany, Greece, Hungary, Ireland, Italy, Latvia, Lithuania, Luxembourg, Netherlands, Poland, Portugal, Romania, Slovakia, Slovenia, Spain, Sweden) and the United Kingdom at a temporal resolution of one hour (Table 3). Note that a zero-congestion level means the traffic is fluid or ‘normal’ but does not mean there are no vehicles and zero emissions. The lower threshold of emissions when the congestion level is zero was estimated using real-time data from an average of 70 main roads in the city of Paris. The daily mean car counts (called Q hereafter) were calculated by a sigmoid function based on regression:

$$Q=a+\frac{b{G}^{c}}{{d}^{c}+{G}^{c}}$$

Where a, b, c, d are regression parameters. Then we allocated the country-level emissions from EDGAR into a daily scale using:

$$Emi{s}_{groundtransport,d}=Emi{s}_{groundtransport,yearly}\times \frac{{Q}_{d}}{{\sum }_{d=1}^{n}{Q}_{d}}$$

where Q_d_ is the mean vehicle number per hour in day d, Emis_ground transport,d_ is the ground transport emission in day d, Emis_ground transport,yearly_ is the annual road transportation emissions from EDGAR, n is the number of days in a year. For countries not covered by TomTom (Croatia, Cyprus, Malta), we assume that their emission changes follow the patterns of total daily emissions from the average other 24 EU countries and the United Kingdom.

**Table 3:** Cities (203 across 24 EU countries and UK) where TomTom congestion level data are available.

Country/Region	Cities with TomTom data
Austria (5)	Vienna, Salzburg, Graz, Innsbruck, Linz
Belgium (10)	Brussels, Antwerp, Namur, Leuven, Ghent, Liege, Kortrijk, Mons, Bruges, Charleroi
Bulgaria (1)	Sofia
Czech Republic (3)	Brno, Prague, Ostrava
Denmark (3)	Copenhagen, Aarhus, Odense
Estonia (1)	Tallinn
Finland (3)	Helsinki, Turku, Tampere
France (25)	Paris, Marseille, Bordeaux, Nice, Grenoble, Lyon, Toulon, Toulouse, Montpellier, Nantes, Strasbourg, Lille, Clermont-Ferrand, Brest, Rennes, Rouen, Le Havre, Saint Etienne, Nancy, Avignon, Orleans, Le Mans, Dijon, Reims, Tours
Germany (26)	Hamburg, Berlin, Nuremberg, Bremen, Stuttgart, Munich, Bonn, Frankfurt am main, Dresden, Cologne, Wiesbaden, Ruhr Region West, Leipzig, Hannover, Kiel, Freiburg, Dusseldorf, Karlsruhe, Ruhr Region East, Munster, Augsburg, Monchengladbach, Mannheim, Bielefeld, Wuppertal, Kassel
Greece (2)	Athens, Thessaloniki
Hungary (1)	Budapest
Ireland (3)	Dublin, Cork, Limerick
Italy (25)	Rome, Palermo, Messina, Genoa, Naples, Milan, Catania, Bari, Reggio Calabria, Bologna, Florence, Turin, Prato, Cagliari, Pescara, Livorno, Trieste, Verona, Taranto, Reggio Emilia, Ravenna, Padua, Parma, Modena, Brescia
Latvia (1)	Riga
Lithuania (1)	Vilnius
Luxembourg (1)	Luxembourg
Netherlands (17)	The Hague, Haarlem, Leiden, Arnhem, Amsterdam, Rotterdam, Nijmegen, Groningen, Eindhoven, Utrecht, Amersfoort, Tilburg, Breda, Apeldoorn, Zwolle, Den Bosch, Almere
Poland (12)	Lodz, Krakow, Poznan, Warsaw, Wroclaw, Bydgoszcz, Gdansk-Gdynia-Sopot, Szczecin, Lublin, Bialystok, Bielsko-Biala, Katowice urban area
Portugal (5)	Lisbon, Porto, Funchal, Braga, Coimbra
Romania (1)	Bucharest
Slovakia (2)	Bratislava, Kosice
Slovenia (1)	Ljubljana
Spain (25)	Barcelona, Palma de Mallorca, Granada, Madrid, Santa Cruz de Tenerife, Seville, A Coruna, Valencia, Malaga, Murcia, Las Palmas, Alicante, Santander, Pamplona, Gijon, Cordoba, Zaragoza, Vitoria Gasteiz, Vigo, Cartagena, Valladolid, Bilbao, Oviedo, San Sebastian, Cadiz
Sweden (4)	Stockholm, Uppsala, Gothenburg, Malmo
UK (25)	Edinburgh, London, Bournemouth, Hull, Belfast, Brighton and Hove, Bristol, Manchester, Leicester, Coventry, Nottingham, Cardiff, Birmingham, Southampton, Leeds-Bradford, Liverpool, Sheffield, Swansea, Newcastle-Sunderland, Glasgow, Reading, Portsmouth, Stoke-on-Trent, Preston, Middlesbrough

#### Aviation sector

Emissions of the aviation sector separated into domestic and international aviation, are estimated by individual commercial flights data (Table 2) from the Flightradar24 database (https://www.flightradar24.com). We compute CO_2_ emissions by assuming a constant emissions factor of aviation in CO_2_ emissions per km flown, called EF_aviation_ across the whole fleet of aircraft (regional, narrowbody passenger, widebody passenger and freight operations) as the share of flight types has not significantly changed since 2019. The aviation sector separates domestic flights and international flights departing from countries considered in this study. The flights within EU but different countries are considered as international. The daily emissions of the aviation sector are estimated as:

$$Emi{s}_{aviation,daily}=DKF\times E{F}_{aviation}$$

where DKF is the distance flown that is computed using great circle distance between the take-off, cruising, descent and landing points for each flight and are cumulated over all flights. For countries with overseas territories (mainly France, UK, Denmark) aviation emissions of flights to/from overseas territories are counted in this study as international emissions, whereas they would be reported as domestic emissions by national estimates.

#### Residential sector

We use the fluctuation of air temperature (Table 2) to simulate daily variations in the energy consumption and direct emissions of residential and commercial buildings. The calculation of residential emissions was performed in three steps: (1) Calculation of population-weighted heating degree days each day for each country of EU27 & UK and for each day based on the ERA5 reanalysis of 2-m air temperature^36,37^, (2) Using annual residential emissions in 2019 from EDGAR as the baseline. For each country, the residential emissions were split into two parts, i.e., cooking emissions and heating emissions, according to the EDGAR guidelines. The emissions from cooking were assumed to remain stable, while the emissions from heating were assumed to depend on and vary by the heating demand. (3) Based on the change of population-weighted heating degree days in each country, we scaled the EDGAR 2019 residential emissions to 2020 and 2021. Since the index of heating degree days are daily values, we get daily emission updates for the residential sources. The EDGAR residential emissions are downscaled to daily values based on daily variations in population-weighted heating degree days as follows:

$$Emi{s}_{residential,monthly}=Emi{s}_{residential,yearly,2019}\times \frac{{\sum }_{m}HD{D}_{daily}}{{\sum }_{m,2019}HD{D}_{daily,2019}}$$

$$\begin{array}{l}Emi{s}_{residential,daily}=Emi{s}_{residential,monthly}\times Rati{o}_{heating,monthly}\times \frac{HD{D}_{daily}}{{\sum }_{m}HD{D}_{daily}}\\ +Emi{s}_{residential,monthly}\times \left(1-Rati{o}_{heating,monthly}\right)\times \frac{1}{{N}_{m}}\end{array}$$

$$HD{D}_{daily}=\frac{\sum \left(Po{p}_{grid}\times \left({T}_{grid,daily}-18\right)\right)}{\sum Po{p}_{grid}}$$

Where *m* is the month, *HDD*_*daily*_ the population-weighted heating degree day, *Ratio*_*heating,monthly*_ the percentage of residential emissions from heating demand monthly, *N*_*m*_ the number of days in month m, *Pop*_*grid*_ the gridded population data derived from Gridded Population of the World, Version 4^36^, *T*_*grid,daily*_ is the daily average air temperature at 2 meters derived from ERA5^37^, and 18 is a HDD reference temperature of 18 °C following ref. ^38^.

### Code description

Python code for data generation is provided (link in the Code Availability section). The codes in CM_EU_v2.py produce daily emissions for 27 EU countries and the United Kingdom in the dataset.

## Data Records

The CM-EU dataset is an CSV file containing country-level emission of 27 European Union countries and the United Kingdom from 01/01/2019 to 31/12/2021 for six sectors: power, industry, ground transportation, domestic and international aviation. The dataset comprises five columns: country, date, sector, value, and timestamp. The value column denotes the daily CO_2_ emissions in Mt CO_2_. The timestamp is included to help servers recognize time. At the time of writing this article, this dataset has been updated to December 31, 2021, and the full dataset can be downloaded at Figshare^34^. Latest updates and related information are available for view and download on our website https://eu.carbonmonitor.org/.

The power sector emissions for Lithuania, Luxembourg, Malta, Romania, and Sweden, in addition to ground transport emissions for Croatia, Cyprus, and Malta, have been estimated by assuming a linear relationship with the daily emissions totals from the remaining countries in their respective sectors. This estimation method was employed due to the lack of coverage or incorrect data provided by the activity data.

**Fig. 1:**
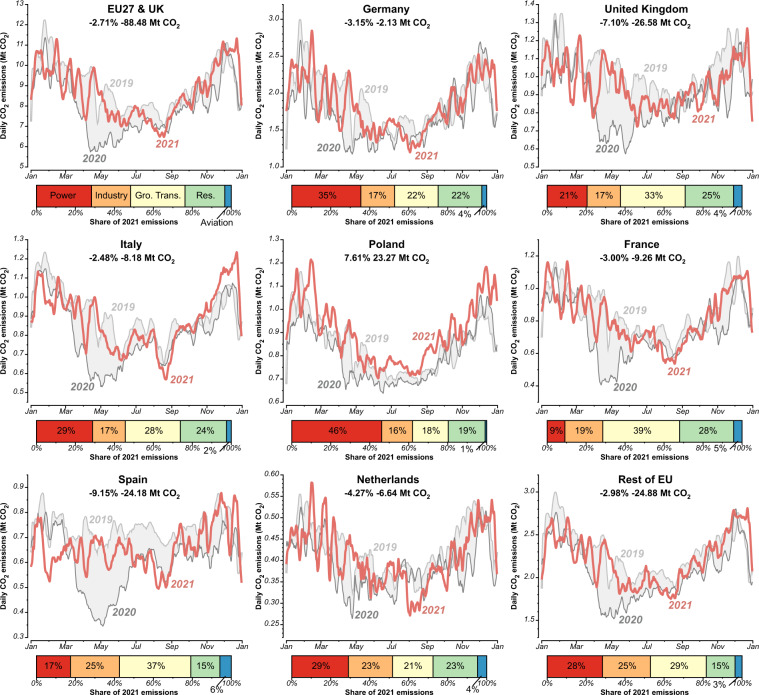
7-days smoothed (running mean) daily CO_2_ emissions for EU27 & UK, Germany, the United Kingdom, Italy, Poland, France, Spain, Netherlands and the rest of European countries in 2019, 2020 and 2021. Grey shaded areas indicate the changes between 2019 and 2020. The percentage and numbers in the top of each panel reflect the relative and absolute change in 2021 compared with 2019. Bars at the bottom show the sectoral shares of annual emissions in 2021. (Aviation includes the domestic and international aviation).

**Fig. 2:**
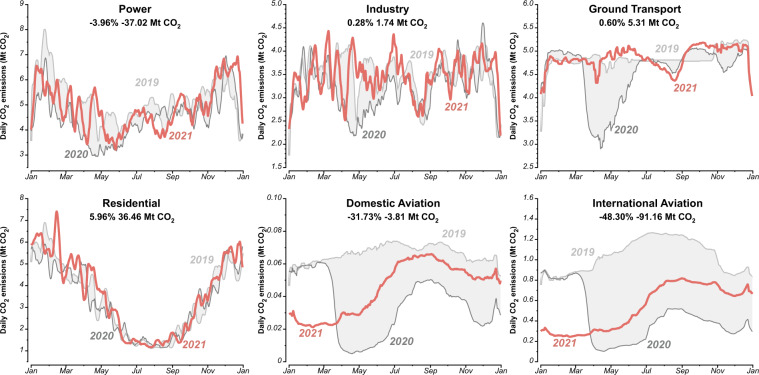
7-days smoothed (running mean) daily CO_2_ emissions for EU27 & UK for six sectors in 2019, 2020 and 2021. Grey shaded areas indicate the changes between 2019 and 2020. The percentage and number in the top of each panel reflect the relative and absolute change in 2021 compared with 2019.

**Fig. 3:**
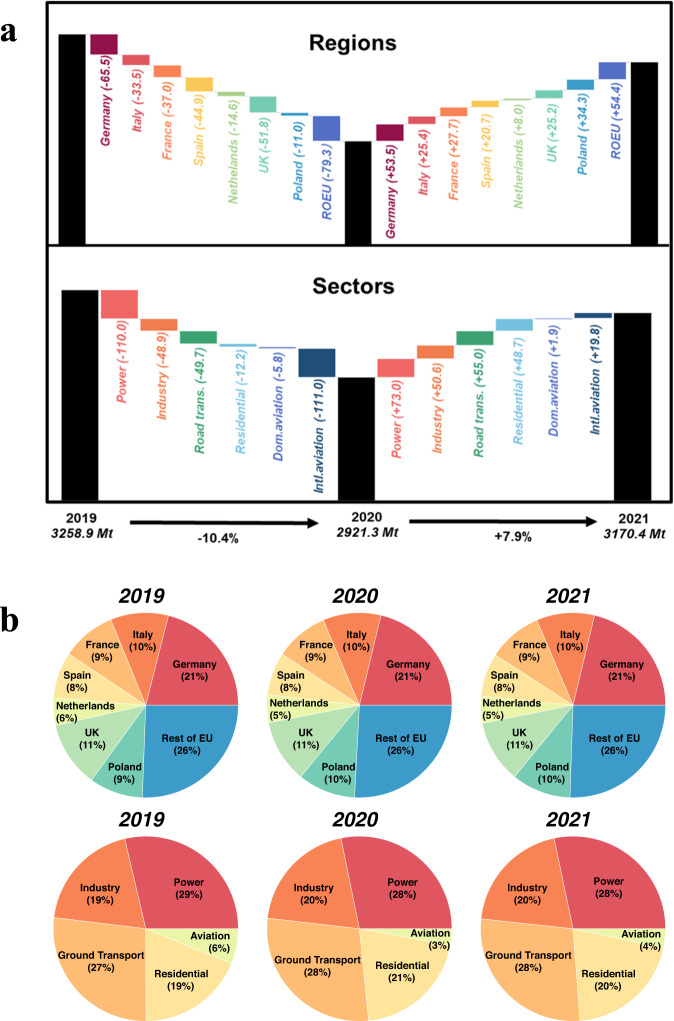
Changes in EU27 & UK emissions from 2019 to 2020 and 2021 across regions and sectors (**a**). Contribution by major countries/emitters and sectors in 2019, 2020 and 2021 (**b**).

**Fig. 4:**
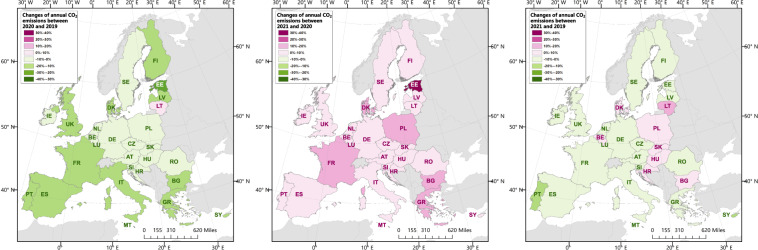
Country-level changes in annual CO_2_ emissions in 2020 compared with 2019 (left), in 2021 compared with 2020 (middle), in 2021 compared with 2019 (right).

Figure 1 shows daily CO_2_ emissions for the years 2019, 2020, and 2021 in the EU27 & UK, as well as for specific countries including Germany, the United Kingdom, Italy, Poland, France, Spain, the Netherlands, and other European countries. Figure 2 represents the daily CO_2_ emissions for six sectors within the EU27 & UK from 2019 to 2021. Figure 3 shows the changes in emissions within the EU27 & UK, from 2019 to 2020 and 2021, across regions and sectors. It highlights the contributions made by major countries and sectors during the study period. Figure 4 shows changes in annual CO_2_ emissions at country level, making comparisons between 2020 and 2019, as well as between 2021 and 2020, and 2021 and 2019 with other published datasets.

## Technical Validation

### Comparison with national emissions estimates, yearly and sub-yearly in selected countries, and with other near-real-time estimates

To evaluate the CO_2_ emissions data from CM-EU, we compared our results with three different existing emissions estimates of various timescale (Table 4). The first evaluation data are annual country-level CO_2_ emissions of EU27 & UK in 2019 and 2020 from EDGAR^8^, BP^7^, IEA^39^, GCP^2^ and Eurostat^40^. The second evaluation data are preliminary emissions publicly disclosed on a monthly or quarterly scale with a latency of a few months to one year by national statistics offices/inventory agencies of Netherlands, Sweden, UK, France and Germany, which are from Centraal Bureau van Statistiek^16^, Statistics Sweden^17^, gov.uk^15^, Citepa^14^ and Umwelt Bundesamt^13^ respectively. The third evaluation data include four data sources: annual CO_2_ emissions reported to the UNFCCC^6^, near-real-time daily and monthly CO_2_ estimates of EU from CICERO (updated from ref. ^41^) and Centre of Research on Energy and Clean Air (CREA)^42^, and daily country-level CO_2_ emissions of EU countries and UK from 2019 to 2021 from the Carbon Watch data of Kayrros^20^ focusing on regulated sectors (ETS).

**Table 4:** List of CO_2_ emissions datasets used for technical validation of CM-EU products.

Dataset	Spatial coverage	Temporal coverage	Scope
EDGAR^8^	Global	1970–2020, annual	Fossil fuel use (combustion, flaring), industrial processes (cement, steel, chemicals and urea) and product use
BP^7^	Global	1965–2020, annual	Fossil fuel combustion
IEA^39^	Global	1960–2020, annual	Fossil fuel combustion
GCP^2^	Global	1750–2020, annual	Fossil-fuel burning, cement production, and gas flaring
Eurostat^40^	European Union	1995–2020, annual	all Nomenclature of Economic Activities (NACE activities)
Kayrros Carbon Watch^20^	European Union and the UK	2019–2021, daily	Power generation, heavy industry, ground transportation and aviation
Centraal Bureau van Statistiek (CBS)^16^	Netherlands	2019–2021, quarterly	Electricity, manufacturing, transport, agriculture, buildings and construction
Statistics Sweden (SCB)^17^	Sweden	1990–2020, annual	Territorial CO_2_ emissions according to IPCC category
gov.uk^15^	UK	1990–2020, quarterly	Territorial CO_2_ emissions of energy supply, business, transport, public, residential and other sectors
Citepa^14^	France	2019–2021, monthly	Territorial CO_2_ emissions of energy, industry, agriculture and transports
Umwelt Bundesamt (UB)^13^	Germany	1990–2021, annual	Territorial CO_2_ emissions
CICERO (updated from ref. ^41^)	European Union	2008–2021, monthly	Fossil fuel combustion
CREA^42^	European Union	2016–2021, daily	Fossil fuel combustion

**Fig. 5:**
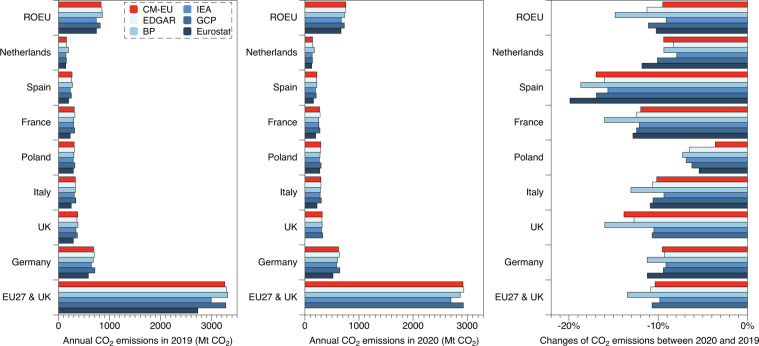
Comparison of annual CO_2_ emissions in 2019 and 2020 and the relative changes between the two years among six datasets: CM-EU, EDGAR, BP, IEA, GCP and Eurostat for EU27 & UK, Germany, UK, Italy, Poland, France, Spain, Netherlands and the rest of EU.

**Fig. 6:**
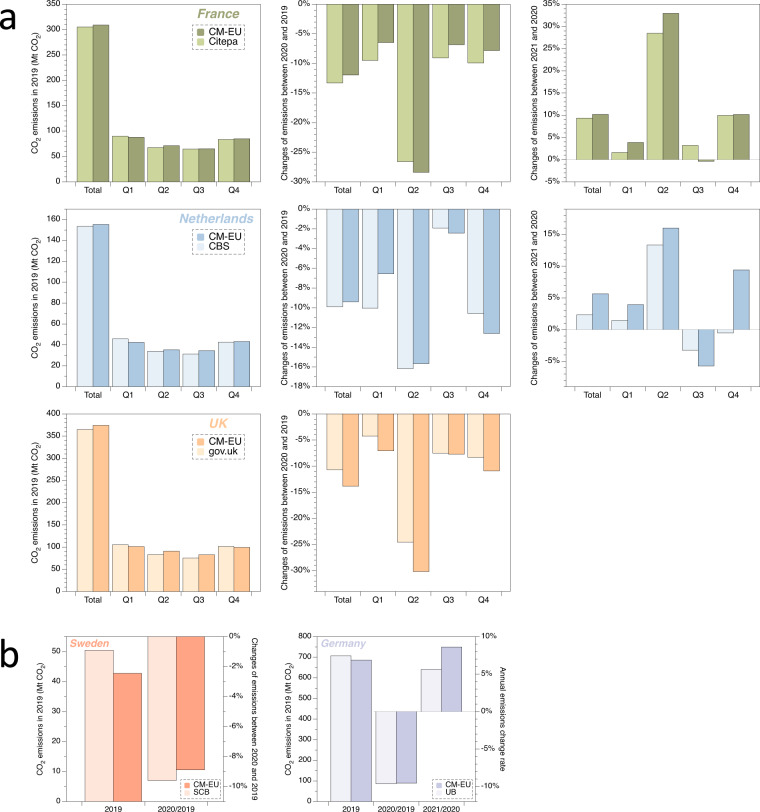
Comparative analysis of quarterly (**a**) and annual (**b**) CO_2_ emissions between CM-EU (darker color) and preliminary national CO_2_ emissions estimates (light color) for France, Netherlands, UK, Sweden and Germany (2019–2021).

Figure 5 shows the comparison of annual CO_2_ emissions in 2019 and 2020 and changes between the two years for EU27 & UK, Germany, UK, Italy, Poland, France, Spain, Netherlands and the rest of EU between CM-EU, EDGAR, BP, IEA, GCP and Eurostat. In terms of annual total amount, the results of the six databases are relatively similar, and the main difference comes from the scope difference of each dataset. As for changes between 2019 and 2020, our estimates are lying in the middle range, close to most other datasets. Figure 6 shows the comparison of annual or quarterly CO_2_ emissions in 2019 and changes between 2020 and 2019 or 2021 and 2020 for Netherlands, UK, Sweden and Germany between this study and estimates from Citepa (France), Centraal Bureau van Statistiek (Netherlands), gov.uk (UK), Statistics Sweden (Sweden) and Umwelt Bundesamt (Germany). It illustrates that our estimates are close to the official estimates in terms of annual or quarterly totals for 2019 and for changes between 2019 and 2020 (at the beginning of COVID-19 pandemic), with relative differences from 0.07% to 3.17% at annual scale and 0.2% to 5.63% at quarterly scale.

Our estimates differ more (in terms of relative differences) from these official estimates for quarterly and annual changes between 2020 and 2021 than between 2019 and 2020, with relative differences from 0.81% to 3.34% at annual scale and 0.19% to 9.94% at quarterly scale.

**Fig. 7:**
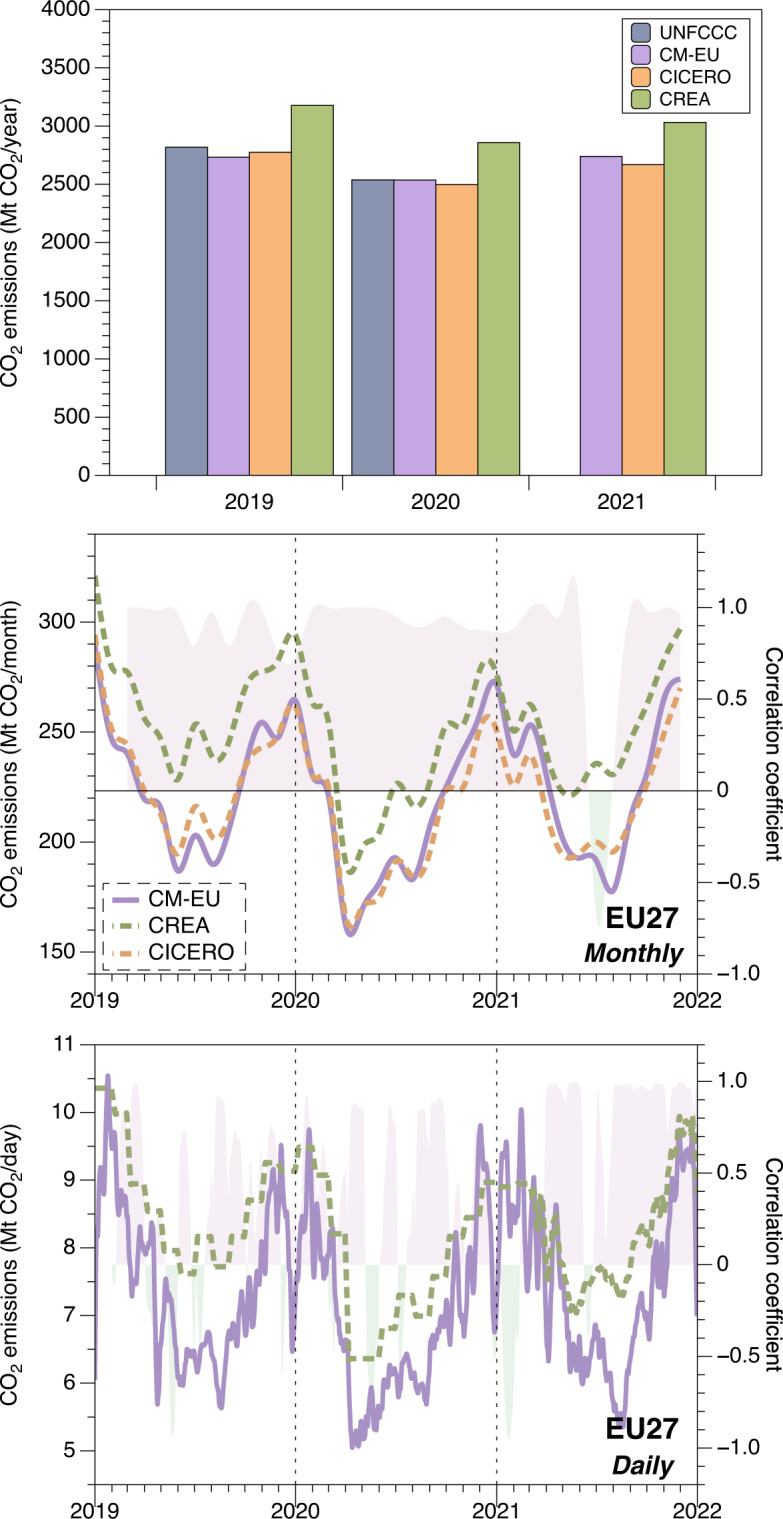
Annual, monthly and daily CO_2_ emissions in 27 EU countries from 2019 to 2021: comparing official reporting to the UNFCCC (dark blue), CM-EU (purple), CREA (green), and CICERO (orange) for annual and monthly data, and CM-EU and CREA for daily data using a seven-day running mean. The shaded area in the line chart represents the correlation coefficient using moving window correlation analysis. Lower daily correlations with CREA before March 2021 are because CREA data are not real daily data in this period (see text).

In Fig. 7, we compare CO_2_ emissions across 27 EU countries from 2019 to 2021, utilizing yearly, monthly, and daily time-steps with other estimates with CREA and CICERO. We omitted the CM-EU emissions from the international aviation sector and UK emissions due to the absence of extra-territorial and UK emissions data in CREA and CICERO. Although CM-EU and CREA offer daily estimates, we averaged them into monthly steps for an easier visual comparison. For monthly data, the emission estimates from all three sources are relatively similar, with CREA reporting a slightly higher emission level. We conducted a moving window correlation analysis of these two sets of data with CM-EU and averaged the results. As can be seen from the shaded portion of the line graph, there is substantial agreement between CM-EU and the other two data sets for the majority of the time, with the exception of July 2021, where a discrepancy was observed. For daily data, we employed a seven-day moving average to CM-EU and CREA emissions. Judging by the trend and shaded portion of the moving window correlation, the two sets of data exhibit a significant level of consistency, with R^2^ values greater than 0.6 occurring approximately 46% of the time. However, the CREA data for 2019 and 2020 are not truly daily, resulting seesaw variability pattern, explaining the difference with CM-EU during these years. Beginning in March 2021, CREA started to provide real daily data, which led to a very high consistency with CM-EU throughout the remainder of the year.

**Fig. 8:**
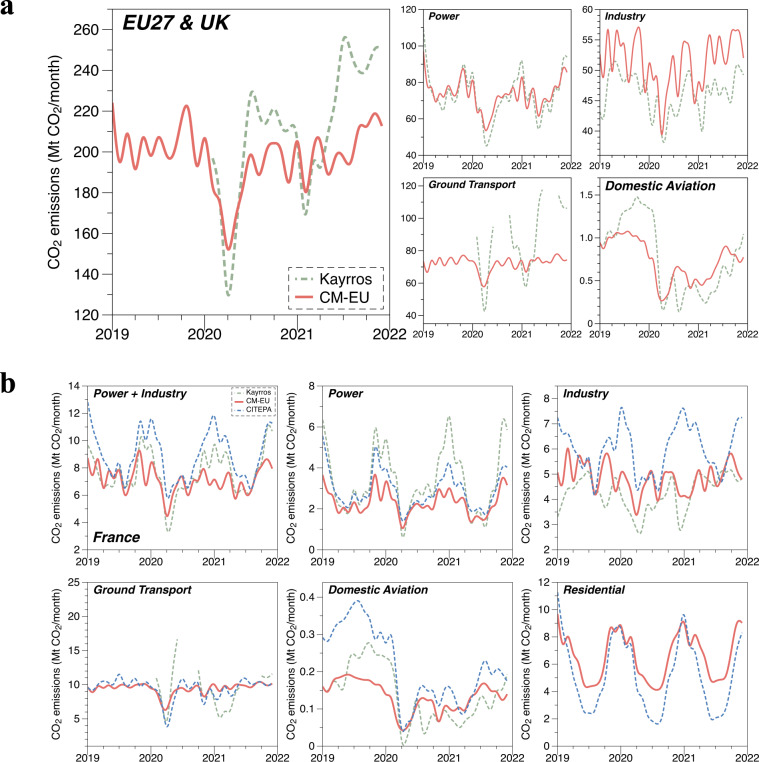
Technical validation of short-term emissions changes with other available ‘high frequency’ data. (**a**) Comparison of monthly CO_2_ emissions for four sectors (power, industry, ground transport and domestic aviation) for EU27 & UK from 2019 to 2021 between CM-EU and Kayrros Carbon Watch data^20^. (**b**) Comparison of monthly CO_2_ emissions in France for power, industry, ground transport, domestic aviation and residential from 2019 to 2021 among CM-EU, Kayrros Carbon Watch data^20^ and CITEPA monthly emission bulletin^14^.

We also compared the monthly CO_2_ emissions of EU27 & UK and France for four or five sectors (power, industry, ground transport, domestic aviation and residential) from 2019 to 2021, with the Kayrros Carbon Watch data and the official Citepa in Fig. 8.

For domestic aviation, we found that CM-EU emissions are lower than Kayrros both in EU27 & UK and France in 2019 (Fig. 8). A possible reason for this difference is that our definition of domestic flights in France only includes trips within metropolitan France, while Kayrros includes flights from France to EU countries and from metropolitan France to French overseas territories. This broader inclusion in Kayrros definition explains why their emissions are larger. The same difference in definitions applies to EU27 & UK. However, it is worth noting that Kayrros estimates for domestic aviation became lower than CM-EU after the outbreak of the COVID-19 pandemic. One possible reason for the lower estimates of domestic aviation emissions by Kayrros compared to CM-EU after the COVID-19 pandemic outbreak is that Kayrros took into account a passenger load factor in their estimates, while CM-EU did not. It is important to note that the passenger load factors of flights have significantly dropped since the COVID-19 pandemic^43^, leading to lower emissions estimates by Kayrros. The reason why Citepa emissions are higher than CM-EU and Kayrros for domestic aviation (Fig. 8) is because the definition of domestic flights for Citepa is all flights in and out of metropolitan-France (mainland), and half of flights between Mainland and French overseas territories (the other half is considered of the responsibility/accounting of the overseas territories). Non-commercial flights are also considered for the estimates of Citepa, which are not in CM-EU and Kayrros.

For industry, the CM-EU emissions are higher than Kayrros, but lower than Citepa. A possible reason for this is the scope of Kayrros covering the regulated industrial installations is lower than the total industry sector of CM-EU, and there are the balances of attributions between power and industry for power production on industrial sites in Kayrros. While all the manufacturing industries, construction and cement production are considered in CM-EU and Citepa. Citepa also considers other industrial productions.

For power, the CM-EU emissions in EU27 & UK are almost the same as Kayrros but small differences in winter peak and summer trough (Fig. 8). A possible reason for this is both datasets use the electricity production by fuel type as activity data from the same data sources. Kayrros can catch the differences from fuel burning efficiency in facility level while CM-EU can’t. But the differences canceled each other when aggregating data for all countries, though they used different annual power emissions as baseline and different emission factors. In France, CM-EU only consider the emissions from power generation in the ‘power’ sector. While urban heating and other electricity self-producers industries are considered in Citepa. Thus, Citepa emissions for ‘power’ are larger than CM-EU. While Kayrros used the annual power emissions from individual French ETS plants annual reporting as the baseline, which are nearly twice larger than those of CM-EU, with largest differences during the cold season. Kayrros also considered the fuel burning efficiency in facility level. This indicates that the aggregate ‘emission factor’ based on 2019 data from EDGAR in CM-EU is lower than the emission factors deduced by Kayrros from ETS declared plant level emissions, a difference that deserves further investigation in the future. Thus, the data of Kayrros have almost the same temporal pattern but are larger with the CM-EU. Due to the different splits of power and industry sector, we add the sum of power + industry for France in Fig. 8b to show that the sum of emissions is in better agreement.

For ground transport, the CM-EU emissions are almost the same than those of the Citepa, but the Kayrros emissions are larger in the summer and lower in the winter. The mobility from trains, expected to increase in the vacation period, was not filtered from vehicles in Kayrros, so the data in the period from July to September were removed.

For residential emissions, the CM-EU emissions are higher in the summer than Citepa. This may be because we assume that the residential emissions in the summer are equal to the emissions from cooking, and cooking emissions were assumed to remain stable all the year, while the emissions from heating were assumed to depend on and vary by the heating demand.

### Technical validation for ground transportation sector and residential sector

**Fig. 9:**
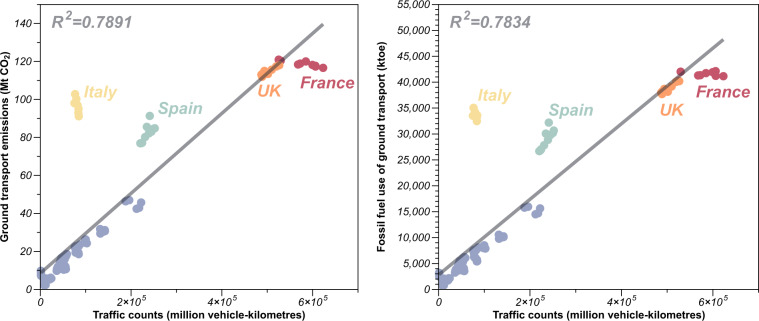
Correlation of annual ground transportation traffic counts with CO_2_ emissions and fossil fuel use in EU countries and the United Kingdom.

In the ground transport sector, we assumed a linear relationship between traffic counts data and emissions or fossil fuel consumption, which assumes that large scale congestion (emissions per vehicle depend on their speed) and vehicle fleets types and emission factors have not changed significantly. To validate these assumptions, we compared annual ground transportation traffic counts with CO_2_ emissions and fossil fuel use in EU countries and the United Kingdom from 2010 to 2019 in Fig. 9. The annual traffic counts data we used come from Eurostat^44^, defined as motor vehicle movements on national territory (irrespective of registration country), covering the United Kingdom and EU countries except Denmark, Germany, Greece, Luxembourg, Malta, Portugal and Slovakia. The annual fossil fuel use of ground transport sector data come from IEA^45^, including four types of fossil fuel (coal, crude oil, oil products and natural gas). The CO_2_ emissions data of ground transport are from EDGAR^8^, including road transportation, rail transportation, inland navigation and other transportation. The comparison statistics shown in Fig. 9 indicate that the coefficient of determination (R^2^) values are 0.7891 between traffic counts and CM-EU ground transport emissions and 0.7834 between traffic counts and fossil fuel use of ground transport, respectively. These findings affirm the assumption’s validity for the EU region and interregional shifts.

For the residential sector, only temperature is assumed to cause variation of emissions in our approach. Ciais *et al*.^46^ looked at changes of direct natural gas use in the residential sector for few European countries from ENSTO-G pipeline data during the first half of the year 2020 and found that temperature variations indeed dominated the observed changes despite the confinements in the cold season, excepted for France and Italy where an additional reduced consumption independent of temperature was found during the weeks of strictest confinements. Here we compared daily emissions from CM-EU with estimates derived from natural gas use from ENSTO-G^47^ in Austria, Belgium_Luxembourg (Belgium and Luxembourg combined), Bulgaria, Croatia, Denmark_Sweden (Denmark and Sweden combined), Finland, France, Germany, Greece, Hungary, Ireland, Italy, Latvia_Estonia (Latvia and Estonia combined), Netherlands, Poland, Portugal, Romania, Slovakia, Slovenia and United Kingdom. Here we use natural gas use data from ENTSO-G as activity data instead, and annual residential emissions in 2019 from EDGAR as baseline. The average yearly emission factor of the whole residential sector is assumed to remain constant at its 2019 value, so that daily emissions are estimated as:

$$Emi{s}_{residential,daily,2019}=Emi{s}_{residential,yearly,2019}\times \frac{A{D}_{residential,daily,2019}}{A{D}_{residential,yearly,2019}}$$

$$Emi{s}_{residential,daily,2020or2021}=Emi{s}_{residential,daily,2019}\times \frac{A{D}_{residential,daily,2020or2021}}{A{D}_{residential,daily,2019}}$$

**Fig. 10:**
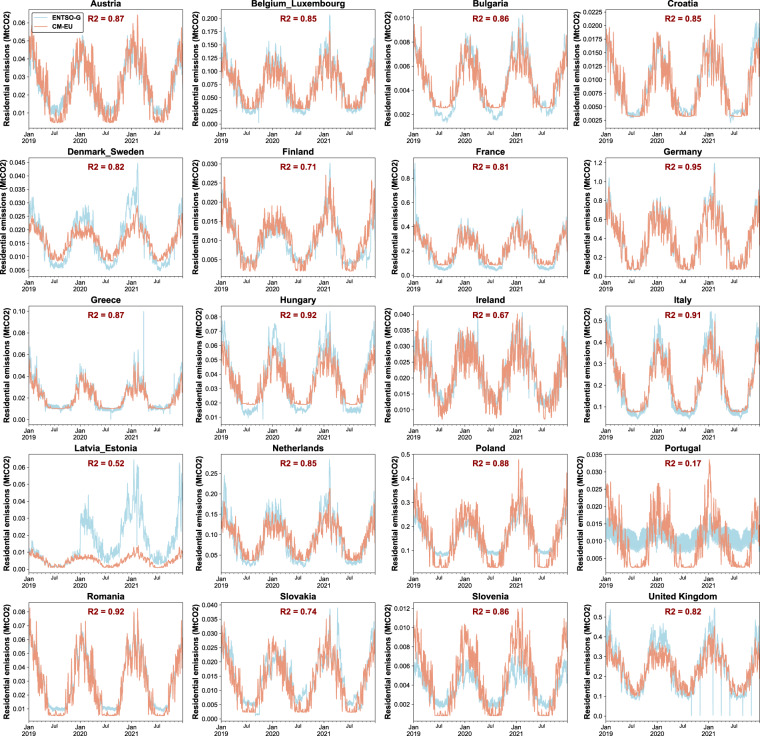
Comparison of daily CO_2_ emissions of residential sector from 2019 to 2021 between CM-EU and ENTSO-G.

**Fig. 11:**
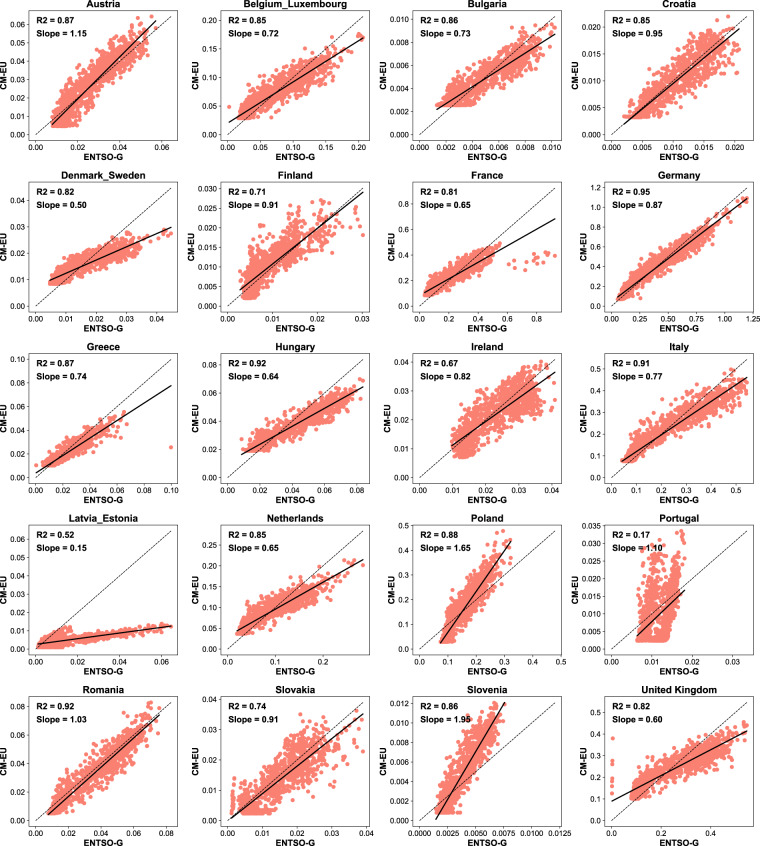
Correlation between CM-EU residential emissions and estimates derived from natural gas use from ENSTO-G.

The ENTSO-G consumption data was completed with Trading Hub Europe (THE)^48^ for the German and e-control^49^ for Austria. Note that the consumption sectors are not provided by e-control dataset. We further split the consumption into more detailed sectors, including household and public buildings heating, industry, and others based on energy balance datasets from Eurostat^50^ following the method from Zhou *et al*.^51^ The activity data used here are only from gas fuel of household and public heating, and we assume that there is a linear relationship between gas fuel use and total fossil fuel use for residential sector. The results in Figs. 10, 11 show that the variation of residential emissions in our study are similar to those of ENTSO-G in most countries (R^2^ ranges from 0.67 to 0.95). Latvia_Estonia (R^2^ = 0.52) and Portugal (R^2^ = 0.17) stand out with significant differences in their residential emissions data compared to the ENTSO-G results. A possible reason is that the variations of coal and petroleum uses for residential heating are not considered in the activity data from ENTSO-G. Therefore, in certain countries where coal and oil are extensively used in residential sector, gas consumption data may not be indicative of the overall energy use patterns in the residential sector.

### Uncertainty analysis

There are two main sources of uncertainties in the CM-EU data. 1. The uncertainty inherited from the EDGAR annual national emissions used for the reference year 2019. 2. Uncertainty from daily activity data and models used to downscale them into daily emissions. The uncertainty analysis was conducted based on the 2006 IPCC Guidelines for National Greenhouse Gas Inventories^35^ and the methodology outlined in the global Carbon Monitor^1,21^. First, uncertainties were calculated for each sector of CM-EU.

For the power sector, CM-EU uses daily statistics of actual thermal production as activity data. When no uncertainty information is available, the 2-sigma uncertainty of the power activity data is assumed to be ±5% according to the IPCC recommended default uncertainty range of energy statistics^35^. In addition, for emission factors, the uncertainties mainly come from the variability of coal emission factors (as coal has a wide range of emission factors of different coal types) and changes in the mix of fuels in thermal power production. CM-EU calculates emission factors based on annual thermal production^7^ and annual power emissions^8^, and the uncertainty range is ±13%. We used error propagation equations to combine the aforementioned uncertainties of each part and estimated the uncertainties of annual power emissions as ±10%.

For the industry sector, a 2-sigma uncertainty (±36%) of CO_2_ emissions from industry and cement production is estimated from monthly production data and sectoral emission factors. The uncertainty of industrial output data is assumed to be ±20% in the industry sector^52^. For the sectoral emission factor uncertainty, according to Carbon Monitor methodology, we calculate national emission factors in 2010–2012 in USA, France, Japan, Brazil, Germany, and Italy according to data availability of monthly emission data and IPI data, and their 2-sigma uncertainties vary from ±14% to ±28. Thus, we adopt a conservative uncertainty of ±30% for CM-EU emissions in this sector.

For the ground transport sector, the global Carbon Monitor methodology assessed a 2-sigma uncertainty of ±9.3% from the prediction interval of the regression model built in Paris to estimate the emissions from this sector. Note that the regression model in Paris between car counts and the TomTom congestion index was based on assuming a relative magnitude in car counts; thus, emissions follow a similar relationship with the TomTom congestion index in Paris.

For the residential sector, global Carbon Monitor compares the estimates by using our methodology with estimates from publicly available natural gas daily consumption data by residential and commercial buildings for France (https://www.smart.grtgaz.com/fr/consommation). The 2-sigma uncertainty of the daily emission estimations is further quantified as ±40%.

For the aviation sector, global Carbon Monitor compares estimates by using two different activity data, i.e., the flight route distance (what we used in this study) and the number of flights and calculate the average difference to quantify the uncertainty of ±10.2% in the aviation sector.

Overall, the uncertainty ranges of the power, ground transportation, industry, residential, and aviation are ±10.0%, ±9.3%, ±30.0%, ±40.0%, and ±10.2%, respectively and the uncertainty in the emission of EDGAR for 2019 is estimated as ±7.1%^53^.

Then, we combine all the uncertainties by following the error propagation equation.

$${U}_{total}=\frac{\sqrt{\sum {U}_{s}\times {\mu }_{s}}}{\left|\sum {\mu }_{s}\right|}$$

where U_s_ and μ_s_ are the percentage and quantity (daily mean emissions) of the uncertainty of sector s, s respectively. The overall uncertainty is quantified as ±13.6%.

We also make the technical validation for CM-EU. Therefore, the uncertainty is equal to the maximum value between the uncertainty ranges and the mean relative uncertainty from technical validation data. Finally, the overall uncertainty range of CM-EU is estimated as ±13.6%.

## Usage Notes

The generated datasets are available from 10.6084/m9.figshare.20219024.v2. We recommend loading the data with a script that can handle large datasets. Users should also note that the unit of emissions in this dataset is Mt CO_2_. Latest updates and related information are available for view and download on our website https://eu.carbonmonitor.org/.

## Data Availability

Python code for producing data for 27 EU countries and the United Kingdom in the dataset is provided at https://github.com/kepiyu/Carbon-Monitor-Europe/blob/main/CM_EU_v2.py.
